# Epidemiological Profile of Psoriasis and Linked Comorbidities in Chinese Population at Shenzhen: A Cross‐Sectional Study

**DOI:** 10.1002/hsr2.71159

**Published:** 2025-09-16

**Authors:** Yanhua Liang, Ntawuyamara Epipode, Li Deng, Zhenyin Zhang, Fuxi Wang, Lin Dang, Zhengfeng Li, Jianglin Zhang, Hengan Yi, Hao Zhang, Shan Yang, Jialin Yan, Weishan Gan, Dejian Duan, Baoqing Deng

**Affiliations:** ^1^ Department of Dermatology, Cosmetology and Venereology, Shenzhen Hospital Southern Medical University Shenzhen Guangdong P.R. China; ^2^ Department of Dermatology, The Eighth Affiliated Hospital Sun Yat‐sen University Shenzhen Guangdong P.R. China; ^3^ Department of Dermatology Shenzhen Second People's Hospital Shenzhen Guangdong P.R. China; ^4^ Department of Dermatology Longgang Central Hospital Shenzhen Guangdong P.R. China; ^5^ Department of Dermatology The University of Hong Kong‐Shenzhen Hospital Shenzhen Guangdong P.R. China; ^6^ Department of Dermatology Shenzhen People's Hospital Shenzhen Guangdong P.R. China; ^7^ Department of Dermatology The Third People's Hospital of Longgang District Shenzhen Guangdong P.R. China; ^8^ Department of Dermatology Baoan Central Hospital of Shenzhen and the Affiliated Baoan Central Hospital of Guangdong Medical University Shenzhen Guangdong P.R. China; ^9^ Department of Dermatology Shenzhen Yantian District People's Hospital (Group) Shenzhen Guangdong P.R. China; ^10^ Department of Dermatology University of Chinese Academy of Sciences–Shenzhen Hospital Shenzhen Guangdong P.R. China; ^11^ Department of Dermatology Dapeng New District Kuichong People's Hospital Shenzhen Guangdong P. R. China; ^12^ Department of Dermatology Shenzhen Bao'an Chinese Medicine Hospital Guangzhou University of Chinese Medicine Shenzhen Guangdong P.R. China; ^13^ Department of Dermatology Shenzhen Baoan Center for Chronic Disease Control Shenzhen Guangdong P.R. China

**Keywords:** body mass index, China, comorbidity, epidemiology, psoriasis

## Abstract

**Background and Aims:**

Only 19% of countries have epidemiological data on psoriasis while an increasing trend in psoriasis prevalence has been consistently reported. The aim of this study is to assess psoriasis epidemiological profile and associated co‐morbidities in the largest immigrant city of Shenzhen in China.

**Methods:**

We conducted a multicenter population‐based cross‐sectional study of 1000 patients from 13 hospitals with dermatology department localized in Shenzhen. Diagnosis of psoriasis was confirmed by experienced dermatologist after physical examination and additional screening as needed. Data were filled in an online form which was completed after scan of a QR code by every patient. Analysis with Excel and SPSS software included patients diagnosed with psoriasis from March 2022 to January 2023.

**Results:**

In total, there were 626 males and 374 females with an average age of 36.39 years. The mean Body mass index (BMI) was 27.0 ± 9.8 SD and high in males (28.18 ± 9.6 SD) compared to females (25.2 ± 9.8 SD) with a statistically significant difference (*p* < 0.001). Psoriatic patients (51.3%) had BMI higher than the Chinese population's normal range; 24.5% of patients being obese. Only 171 patients had a psoriasis family history (17.1%). The age of onset was between 11 and 40 years old, accounting for 80% of the total patients. About 36.7%, 37.1% and 26.2% cases present mild, moderate, and severe cutaneous conditions, respectively. Among the patients, 64.7% presented at least one kind of comorbidities, predominantly rheumatic diseases (48.7%), endocrine system diseases (29.8%) and nervous system diseases (24.4%). The proportion of comorbidities in the mild, moderate and severe groups was 59.9%, 59.0%, and 79.3% respectively.

**Conclusion:**

Psoriasis is more common in young and middle‐aged people. BMI in psoriatic population was higher compared to Chinese common population. More patients presented with moderate to severe psoriasis. More severe psoriasis carries more comorbidities.

## Summary

- Only 19% of countries have epidemiological data on psoriasis, while an increasing trend in psoriasis prevalence has been consistently reported.
- Among the patients, 64.7% presented at least one kind of psoriasis comorbidity, and body mass index in psoriatic population was higher compared to the Chinese common population.
- Clinicians should consider psoriasis a systemic disease and be aware that the more severe the disease, the more comorbidities can occur.

## 1 Background

Psoriasis is a persistent, immune‐mediated, inflammatory systemic disease that is non‐communicable. It mainly affects the skin, nails, and joints [1]. Robert Willan, regarded as the founder of modern dermatology, is acknowledged for providing the first detailed clinical account of psoriasis. As a result, the condition is also referred to as Willan's lepra [2]. The common type is psoriasis vulgaris and is divided into two forms: type 1, which is hereditary with early onset (< 40 years) and type 2, which is sporadic and occurs in older age (> 40 years) [3].

An estimated 60 million people have psoriasis worldwide. In every country, the prevalence of psoriasis in children is less than 1%. Among adults, psoriasis prevalence ranges from 0.17% in East Asia to 2.50% in Western Europe. The prevalence of psoriasis differs according to geographical location [4]. Psoriasis is a multifactorial disease with similar prevalence in both males and females. However, findings from a recent study indicate that, on average, men tend to experience more severe forms of the condition compared to women [5].

In 2019, China reported the highest number of psoriasis cases at 7.65 million (95% uncertainty interval [UI]: 7.39–7.91), followed by India with 4.8 million cases (95% UI: 4.63–4.96). China had the largest prevalence cases, 7.65 million (95% UI: 7.39–7.91), followed by India with 4.8 million (95% UI: 4.63.6–4.96). In China, the age‐specific prevalence of psoriasis cases in 2019 showed a rightward shift compared to 1990, with the highest concentration occurring between the ages of 50 and 54 years. Additionally, there was a noticeable excess of cases in males aged 40 to 69 years. Despite China still having the largest number of psoriasis cases globally in 2019, the growth rate of these cases remained below the global average [6]. According to the Global Psoriasis Atlas (GPA), 2.36 million Chinese people are affected by psoriasis, which makes a prevalence of 0.17% [7].

The most frequent symptoms of psoriasis reported by individuals are scaling of the skin, itching, erythema, fatigue, swelling, burning and bleeding [8]. Many individuals with psoriasis also have serious health conditions like diabetes, heart disease, and depression. Additionally, some people with psoriasis experience an inflammatory joint condition known as psoriatic arthritis (PsA) [9]. Salgado‐Boquete et al. introduced a ranking algorithm that incorporates various disease assessment metrics to categorize psoriatic patients into three groups: mild, moderate, and severe [10].

Psoriasis is frequently associated with different co‐morbidities, including cardiovascular diseases such hypertension (HTA) and cardiac insufficiency, metabolic disorders like diabetes mellitus (DM), dyslipidemia, and metabolic syndrome (MetS), inflammatory bowel disease, kidney disease, and depression. Compared to control groups, patients with psoriasis have higher rates of multiple risk factors, including HTA, obesity, diabetes, dyslipidemia, MetS, and cigarette smoking [11]. Psoriasis has long been recognized as a disease with numerous complications, once thought to be linked to diet and obesity. However, in recent years, psoriasis itself has been identified as a systemic inflammatory disorder, in which the involved cytokines can trigger a variety of other diseases [12].

Only 19% of countries have epidemiological data on psoriasis. Studies worldwide suggested a stable or slightly decreasing trend in psoriasis incidence, while an increasing trend in psoriasis prevalence has been consistently reported [4, 13]. Further research is required to determine the reasons driving the increase in psoriasis prevalence over time. It is also needed to figure out the constitution and frequency of co‐morbidities, respectively, in mild, moderate, and severe psoriatic groups, which will shed light on the perspectives for patient education and making therapeutic decisions.

## 2 Materials and Methods

### 2.1 Study Design

We conducted a multicenter, population‐based and analytical cross‐sectional study of patients from 13 hospitals with dermatology department localized in Shenzhen city, China. Shenzhen is a large, immigrant, fast‐growing city in southern region of China. Analysis included 1000 patients diagnosed with psoriasis from March 2022 to January 2023.

Patient variables collected were sociodemographic data (age, gender, residence) and anthropometric measurement such as height and weight, used to calculate Body Mass Index (BMI). We collected also variables targeting psoriasis disease assessment such as disease onset, course and duration, body sites currently affected, family history of psoriasis and percentage of body surface area (BSA) involved. The type of comorbidity, if any, was mentioned for every patient.

The diagnosis of psoriasis was confirmed by consulting dermatologist after physical examination and additional screening like laboratory and imaging results as required. Data were surveyed in an online form which was completed after scan of a QR code by every patient. The dermatologist also determined the severity of psoriasis using BSA, namely, mild (limited disease with you ≤ 3% BSA affected), moderate (scattered disease with 3%–10% BSA affected), and severe psoriasis (extensive disease with > 10% BSA affected), the formula which has been used by other investigators [14, 15, 16, 17]. BMI was calculated by the formula [18, 19]: weight (kg)/[height (m)]^2^.

### 2.2 Data Collection and Analysis

After a diagnosis of psoriasis was made by a dermatologist; sociodemographic, anthropometric and psoriasis disease related data were filled by every patient who first scanned a QR code to open an online questionnaire translated in Chinese language (see Supporting Information S1: Appendix 1). The form contained all variables and alternative responses. Dermatologists was available to explain and help the patient who didn't understand how to fill the form. After that, consulting dermatologist could fill in the form data from physical examination: psoriasis type, BMI, BSA, psoriasis severity and type of comorbidities. A physician also checked the completeness of the form before submission. Data collected were downloaded in Excel data set which was checked for completeness. Results were presented in tables and figures. Simple analysis, tables and figures were designed using Excel software 2016. We transferred Excel data set to SPSS software 25.0 version for advanced analysis and statistical significance check. Means for BMI were compared using independent *z*‐test. Significance level was fixed at two‐side *p* value below 0.05.

### 2.3 Ethical Clearance

The study has been approved by Shenzhen Hospital of Southern Medical University Review Board (Reference number: CN‐HUMD‐220021). The study was conducted according to the Declaration of Helsinki Principles. Participants gave their written informed consent.

## 3 Results

### 3.1 Psoriasis Is More Prevalent in Young and Middle‐Aged People

From March 2022 to January 2023, 1000 patients have been diagnosed with psoriasis in 13 departments of dermatology across Shenzhen city. Of these, 626 were males (62.6%) and 374 females (37.4%). The age of patients varied from 10 to 85 with the mean age of 36.3 years (see Table 1).

**Table 1:** Epidemiological characteristics including family history and BMI.

Variables	Total	Male	Female	*p* value
Mean age (years)	36.3 ± 11.9	36.7 ± 12.0	35.7 ± 11.7	0.164
Age of onset (years)	27.9 ± 12.2	28.5 ± 11.9	27.0 ± 12.8	0.064
Family history	171	109	62	0.735
Mean BMI (kg/m^2^)	27.0 ± 9.8	28.1 ± 9.6	25.2 ± 9.8	< 0.001

**Figure 1:**
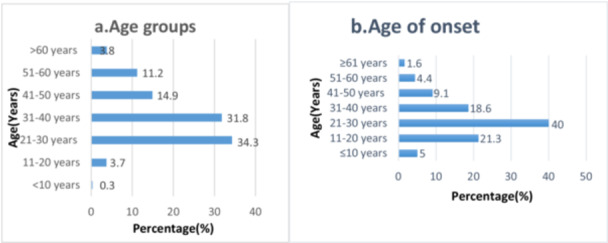
Shows (a) classification of psoriatic patients according to age group and psoriasis age of onset in (b).

Psoriasis patients are mainly young (66.1%) belonging to the age groups of 21‐30 and 31‐40 years old (Figure. 1a). More cases of psoriasis occurred from 11 to 40 years old (80%), a half of them (40%) began to suffer from psoriasis at the age between 21 and 30 years old (Figure 1b).

### 3.2 BMI in Psoriatic Population Was Higher

The mean BMI is 27.0 ± 9.8 SD. Overweight and obesity were represented by 26.8% and 24.5%, respectively. Only 17.1% of psoriatic patients have family history of psoriasis. Comparing psoriatic patients in their gender according to demographic and clinical characteristics, statically significant difference was seen only in BMI (28.1 ± 9.6 SD for male vs. 25.2 ± 9.8 SD for female *p* value < 0.001), where a high mean BMI was found in males (see Table 1). The difference was not statistically significant considering age and psoriasis age of onset between males and females.

### 3.3 Most of Patients Suffer From Moderate or Severe Psoriasis

The moderate form is the most represented with 371 cases followed by the mild form (367 cases) to finish with severe form (262 cases). Nail involvement was seen in 38.9% of the patients, whereas the lesions could be seen on other body sites, like the scalp or other special sites, including the mucous membrane, lesions in the axilla, breast, groin and perineum. Plaque psoriasis was the first represented phenotype, with more than 80.6% taking into account different combined forms like plaque psoriasis with PsA (5.1%), plaque psoriasis with erythrodermic psoriasis (0.9%) and plaque psoriasis with pustular psoriasis (0.4%) (Table 2).

**Table 2:** Clinical features of psoriasis patients.

Clinical features	Number	Percentage (%)
Disease severity		
Mild	367	36.7
Moderate	371	37.1
Severe	262	26.2
Lesions localization		
Finger and toenails	389	38.9
Others localization	537	53.9
Not mentioned	34	3.4
Disease phenotypes		
Plaque psoriasis	806	80.6
Psoriatic arthritis	71	7.1
Erythrodermic psoriasis	42	4.2
Pustular psoriasis	17	1.7
Plaque psoriasis + pustular psoriasis	40	4.0
Plaque psoriasis + erythrodermic psoriasis	90	9.0
Plaque psoriasis + psoriatic arthritis	51	5.1
Total	1000	100

### 3.4 More Severe Is Psoriasis, More Frequent Are Comorbidities

**Figure 2:**
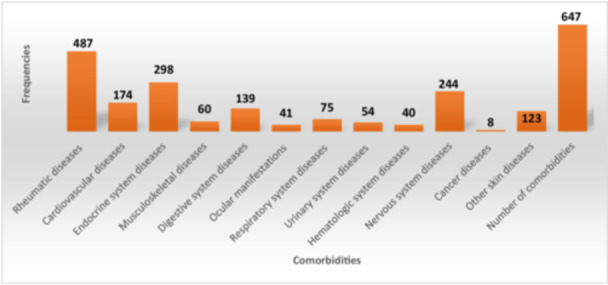
Shows different comorbidities in our study population.

Among the 1000 psoriatic patients, there were 647 patients who have comorbidities, accounting for 64.7%. These are predominantly rheumatic diseases (48.7%), endocrine system diseases (29.8%) and nervous system diseases (24.4%). The less‐seen co‐morbidities are malignant tumors (0.8%) (Figure 2). More details for prevalence of comorbidities are shown in Supporting Information S2: Appendix 2.

**Figure 3:**
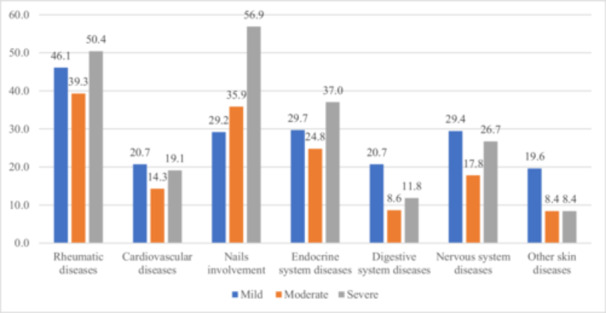
Proportion and trend of main comorbidities in different psoriasis forms.

The proportion of co‐morbidities in mild, moderate, and severe group was respectively 59.9%, 59.0%, and 79.3%. It was higher in severe form compared to other psoriasis severity forms: rheumatic diseases (46.1%), endocrine system diseases (37.0%) and nervous system diseases (26.7%). Nails involvement was seen in more than a half (56.9%) of patients with severe form (Figure 3).

## 4 Discussion

Psoriasis is an autoimmune, chronic disease with different co‐morbidities. It not only affects the skin, but also other systems. This study conducted in the largest immigrant city, Shenzhen, aimed to provide representative psoriasis data and associated co‐morbidities in Chinese population in general.

This study included 1000 psoriatic patients, 626 were males (62.6%) and 374 females (37.4%). Male predominance was similar to that reported from Nepal, Maghreb, Egypt and Malaysia (53.7%, 55.7%, 56.3% and 56.6%, respectively) [20, 21, 22, 23]. For the case of China, this male predominance can also be seen in the general population, which is highly dominated by males [24].

The mean age of patients is 36.3 years old ranging from 10 to 85 years old without significant difference between males and females (*p* = 0.164). Many of psoriatic patients are from 21 to 40 years old age group (66.1%). This is similar to the results found by Mikrani and Shrestha [21] where patients' age ranged from 6 to 86 years (36.1 ± 22.3 SD). El‐Komy et al. found the mean age of 39.3 years in Egypt [22]. Psoriasis mean age of onset in this study is 27.9 ± 12.2 SD, without statistically significant difference between females and males. Most of the patients (80%) showed symptoms of psoriasis between 11 and 40 years old. For Jameel and Arati's study [21], mean age at onset was 26.4 ± 14.3 years (29.7 ± 13.8 SD) in males and 21.4 ± 13.1 SD in females. Queiro et al. [25] highlighted the growing importance of age at disease onset as a key stratification factor in worldwide clinical and genetic studies of psoriatic disease.

A family history of psoriasis was found in only 17.1% of psoriatic patients. Psoriasis has a genetic background. Approximately 40% of patients with psoriasis have a family history of psoriasis or psoriatic arthritis, which may affect disease features [26]. This low percentage of family history in this study may be explained by the low prevalence of psoriasis compared to other parts of the world like Europe and America [4, 27, 28]. Psoriasis age of onset, family history, human lymphocyte antigen type Cw6 (HLA‐Cw6) and disease course are criteria to classify psoriasis in two types [3, 29]. Even if the majority of our study participants (84.9%) had psoriasis onset before 40 years old, the low proportion on family history as well as the lack of results on the other criteria did not allow us to classify our study participants as type one or two.

In this study, plaque psoriasis form was the most represented (80.6%) with combined forms involving it like Plaque Psoriasis and Erythrodermic Psoriasis (9%), Plaque Psoriasis and PsA (5.1%) and Plaque Psoriasis combined with Pustular Psoriasis (4%). It is followed by PsA (7.1%), Erythrodermic Psoriasis (4.2%) to finish with Pustular Psoriasis (1.7%). A study conducted in China showed that erythrodermic psoriasis was reported in only 0.6% of patients. Our results are similar to the study conducted in Egypt [22], where the most prevalent form of psoriasis was chronic plaque type, documented in 84.1% of patients. Less common variants; erythrodermic and pustular psoriasis, were observed in 3.7% and 1.8% of patients, respectively. In a study conducted in China [30] in 2017, the majority of patients (96.5%) had psoriasis vulgaris, while 1.6% had pustular psoriasis, 0.7% had erythrodermic psoriasis, and 1.3% had PsA. Boehncke and Schön stated that chronic plaque psoriasis, also known as psoriasis vulgaris, is the most common form of the disease and constitutes approximately 90% of cases [5]. In the study conducted by Mikrani and Shrestha in Lumbini Medical School, psoriasis vulgaris was the most common type of disease, which accounted for 80% of the cases [21]. In Maghreb, erythrodermic and pustular psoriasis were observed in 13.6% and 5.7% of psoriasis patients, respectively [31]. This difference may be due to the ethnic difference between Chinese population and Maghreb population, which is mainly Arabic. Experts at the Genome University of Singapore (GIS) recently published a report explaining why Caucasians are more susceptible to psoriasis than individuals of Chinese ethnicity [32]. Such diversities may be attributed to a greater number of genetic and epigenetic mechanisms.

Patients were classified into three forms, mild (36.7%), moderate (37.1%) and severe (26.2%). For the purpose of clinical trials, psoriasis may be classified as mild psoriasis, moderate psoriasis, and severe psoriasis [1]. Yeung et al. found that based on BSA criteria, among psoriasis patients, 51.8%, 35.8%, and 12.4% had mild, moderate, or severe disease, respectively [33].

In this study, nails involvement is seen in 38.9% of the patients and more than a half (56.87%) of them were with severe form. Nail psoriasis is a difficult‐to‐treat condition that impacts 50%–79% of individuals with skin psoriasis and as many as 80% of those with PsA [34]. Nail alterations may arise in any form of psoriasis. Among individuals with psoriasis, fingernail changes are observed in 50% of patients, while toenail changes affect 35% of patients [35]. The low percentage seen in our study may be due to the factor that this study was conducted in a developed city where patients consult before the disease gets worse.

The mean BMI in our study is 27.0 ± 9.8 SD. It was higher in males (28.1 ± 9.6 SD) compared to females (25.2 ± 9.8 SD) with a statistically significant difference (*p* value < 0.001). More than half of patients (51.3%) were with weight higher than the Chinese normal range; 24.5% of patients being obese. The BMI found in this study is higher than the standardized mean BMI level in Chinese population: 24·4 kg/m^2^ (24.3–24.6 kg/m²) [36]. This shows a tendency for overweight in psoriatic population. The same tendency was also found by El‐Komy et al. [22] in Egypt, who calculated the mean BMI at 28.0 6 ± 7.3 SD when the mean BMI for the Egyptian population was 28.2. Although the majority of studies have indicated that individuals with psoriasis have substantially greater likelihoods of experiencing obesity [37], other studies have shown a lack of evidence to support this idea [37].

In this study, 64.7% of psoriatic patients had co‐morbidities. These were predominantly rheumatic diseases (48.7.6%), endocrine system diseases (29.8%) and nervous system diseases (24.4%). The less‐seen comorbidities are cancer diseases (0.8%). Major co‐morbidities were in higher proportion in severe form compared to other psoriasis severity forms. For some types of co‐morbidities, many patients had more than one disease like cardiovascular diseases (174 patients with 203 comorbidities).

Association of psoriasis and other autoimmune diseases is a continuing research topic [38]. Our results revealed that psoriasis was associated with other autoimmune diseases such as RA (5.6%), vitiligo (1.3%), systemic sclerosis (2.2%), Crohn's disease (0.8%), celiac disease (0.3%), atopic dermatitis (2.3%), bullous pemphigoid (0.5%), alopecia areata (1.4%), and Hashimoto thyroiditis (2.2%). In addition to psoriasis, our study individuals could present more than one autoimmune disease at the same time. One of the three facts that autoimmune diseases share a common origin is the kaleidoscope of autoimmunity. It is described as the occurrence of multiple autoimmune diseases within a patient or members of a nuclear family [39]. Prior research has demonstrated a greater prevalence of autoimmune diseases among individuals with psoriasis compared to the general population, such as bullous pemphegoid (BP), vitiligo, alopecia areata, thyroiditis, rheumatoid arthritis (RA), Crohn's disease, multiple sclerosis, systemic lupus erythematous (SLE), Sjögren Syndrome and atopic dermatitis [40, 41, 42]. A study conducted on 25,341 psoriatic patients in the United States of America (USA) revealed that 1.5% of them had at least three autoimmune diseases [42].

Other types of co‐morbidities found are endocrine system diseases (298 patients with 380 co‐morbidities) and nervous system diseases (244 patients with 342 co‐morbidities). MetS was the leading disease among endocrine system co‐morbidities (8.2%). Recent studies revealed an independent association of psoriasis and MetS [43, 44]. On the other hand, Kaushik et al. [45] demonstrated that a low level of serum adiponectin is a predictor of severe and prolonged psoriasis, independent of the presence or absence of MetS. A meta‐analysis conducted in 2022 showed a prevalence of MetS of 32% globally and 29% in Asia among psoriatic patients [44]. This prevalence is higher in comparison to our results. The difference may be due to the variation of psoriasis prevalence around different regions of the world and among countries. In other systems like respiratory, hematologic and musculoskeletal, the number of psoriatic patients and co‐morbidities is equal. Other skin diseases found in our psoriatic population are similar to those presented in the population visiting our department, such as pruritus, vitiligo, atopic and contact dermatitis or alopecia. The growing literature across diverse populations and contexts further supports associations between psoriasis and cardiometabolic diseases, gastrointestinal disorders, kidney disease, malignancy, infections, and mood‐related conditions [46]. In this study, rheumatic diseases are a highly represented group of co‐morbidities. They were mainly PsA (12.2%), osteoarthritis (22.4%), axial spondylarthritis (7.2%), rheumatoid arthritis (5.6%) and gout (4.1%). However, prevalence of rheumatic diseases in our study is lower compared to the findings of a study recently conducted in USA to assess the association of psoriasis with rheumatic diseases [47]. More than 80% of psoriatic patients included in the above‐mentioned study had rheumatic diseases; representative diseases included PsA (21.6%), osteoarthritis (37.7%), gout (5.9%) and RA (5.9%). This difference may be due to the difference in epidemiology of psoriasis in China and USA. In this analysis of patients with psoriasis, we can cite among more prevalent comorbidities HTA (10.6%), nonalcoholic fatty liver disease (7.6%), MetS (8.2%), DM (5.0%) and obesity (9.4%), hyperlipidemia (13.2%). Population‐based observational studies have suggested a relationship between psoriasis and HTA [48, 49]. Even if the prevalence of HTA in our psoriatic population is relatively high, it is lower compared to the prevalence found by Kim et al. [49], which was 25%. Certain studies have additionally proposed that individuals with psoriasis are more prone to having more advanced forms of nonalcoholic fatty liver disease (NAFLD) compared to non‐psoriatic control subjects. Moreover, psoriatic patients with NAFLD exhibit more severe psoriasis than those without NAFLD [50]. In a study conducted by Miele et al. [51], the NAFLD prevalence of 59.2% was found in an outpatient cohort of 142 adults with psoriasis. In a real‐world study conducted in USA [52], the prevalence and incidence rates of the most common co‐morbidities were 47.5% and 35.0% for hyperlipidemia, respectively; 47.3% and 31.3% for HTA; 21.2% and 15.4% for depression; 20.2% and 13.5% for type 2 DM; and 16.6% and 12.4% for fibromyalgia. In the study of Husted et al. [53], the prevalence of HTA, obesity, hyperlipidemia, type 2 DM was 37.1%, 30.0%, 20.7%, 12.0%, and 8.2%, respectively. The co‐morbidities in our study were very common in severe form, where 79.3% of psoriatic patients had at least one psoriasis co‐morbid disease, prevalence being the same during mild to moderate forms, 59.95% and 59.03%, respectively. The same idea is shared by Yeung et al. [33], who found that among psoriasis patients, the burdens of overall medical comorbidity and specific comorbid diseases rise as the disease severity increases.

## 5 Limitations

This study shows a high prevalence of comorbidities in the psoriatic Chinese population. However, some shortcomings need to be mentioned: (1) Data were collected at the hospital from people who had just presented psoriasis symptoms, not from community. But also, the data were collected partly during the interaction between participants and physicians and partly through patients' self‐reports. These two factors may impact prevalence of psoriasis and its co‐morbidities in Shenzhen population. A case‐control study to compare the prevalence of psoriasis co‐morbidities in general and psoriatic population, conducted in community with an in‐depth investigation of co‐morbidities and additional screening would be an asset. (2) This study was conducted in different hospitals of Shenzhen, which is an immigrant city. These results can also include data from the non‐Chinese population, even if the percentage may be very low. (3) During our study, we did not search results for HLA‐ Cw6 genotype and psoriasis clinical course, which could help us to confirm whether our participants have psoriasis 1 or 2. Further research with these data is encouraged to classify the Shenzhen psoriatic population.

## 6 Conclusion

Psoriasis is more common in young and middle‐aged people. BMI in the psoriatic population was high, and more than half of them were above the Chinese population's normal range. The prevalence of co‐morbidities is high, and they were mainly rheumatic, endocrine and nervous system diseases. They were more observed in severe form, which requires psoriatic patients to see a dermatologist before the disease worsens.

## Author Contributions

**Yanhua Liang:** conceptualization, supervision, and project administration. **Ntawuyamara Epipode:** conceptualization, writing – original draft, and data curation. **Li Deng:** investigation and validation. **Zhenyin Zhang:** investigation. **Fuxi Wang:** investigation. **Lin Dang:** investigation, writing – review and editing. **Zhengfeng Li:** investigation, writing – review and editing. **Jianglin Zhang:** investigation, writing – review and editing. **Hengan Yi:** investigation and formal analysis. **Hao Zhang:** investigation, writing – review and editing. **Shan Yang:** investigation and formal analysis. **Jialin Yan:** investigation, writing – review and editing, formal analysis. **Weishan Gan:** visualization and resources. **Dejian Duan:** investigation and formal analysis. **Baoqing Deng:** investigation and methodology.

## Conflicts of Interest

The authors declare no conflicts of interest.

## Transparency Statement

The lead author, Yanhua Liang, affirms that this manuscript is an honest, accurate, and transparent account of the study being reported; that no important aspects of the study have been omitted; and that any discrepancies from the study as planned (and, if relevant, registered) have been explained.

## Supporting information


**Appendix 1:** QR code of online questionnaire.

**Appendix 2:** Prevalence of comorbidities observed among psoriasis patients.

## Data Availability

The data that support the findings of this study are available from the corresponding author upon reasonable request.
