# Supplementary figures and images for: Epidemiological Profile of Psoriasis and Linked Comorbidities in Chinese Population at Shenzhen: A Cross‐Sectional Study
Source: Health Sci Rep. 2025 Sep 16;8(9):e71159. doi: 10.1002/hsr2.71159 (PMC12441203; doi:10.1002/hsr2.71159)

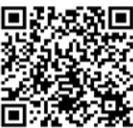


**Appendix 1.** QR code of online questionnaire translated in Chinese

Supplement: Supplementary file 1 — Appendix 1: QR code of online questionnaire. [file HSR2-8-e71159-s001.docx]
