# Supplementary material for: Epidemiological Profile of Psoriasis and Linked Comorbidities in Chinese Population at Shenzhen: A Cross‐Sectional Study
Source: Health Sci Rep. 2025 Sep 16;8(9):e71159. doi: 10.1002/hsr2.71159 (PMC12441203; doi:10.1002/hsr2.71159)
Supplement: Supplementary file 2 — Appendix 2: Prevalence of comorbidities observed among psoriasis patients. [file HSR2-8-e71159-s002.docx]

**Appendix 2. Prevalence of comorbidities observed among psoriasis patients**

| Comorbidity | | Psoriasis population  n=1000 | Number of comorbidities  n (%) |
| --- | --- | --- | --- |
| Rheumatic diseases | | 487(48.7%) | 583(58.3) |
| 1 | Osteoarthritis |  | 224(22.4) |
| 2 | Psoriatic arthritis |  | 122(12.2) |
| 3 | Axial spondylarthritis |  | 72(7.2) |
| 4 | Gout |  | 41(4.1) |
| 5 | Rheumatoid arthritis |  | 56(5.6) |
| 6 | Systemic lupus erythematosus |  | 12(1.2) |
| 7 | Ankylosing spondylitis |  | 16(1.6) |
| 8 | Systemic sclerosis |  | 22(2.2) |
| 9 | Multiple sclerosis |  | 18(1.8) |
| Cardiovascular diseases | | 174(17.4%) | 203(20.3) |
| 1 | Aortic stenosis |  | 37(3.7) |
| 2 | Myocardial infarction |  | 5(0.5) |
| 3 | Stroke |  | 6(0.6) |
| 4 | Pulmonary hypertension |  | 9(0.9) |
| 5 | Peripheral vascular disease |  | 17(1.7) |
| 6 | Ischemic heart disease |  | 5(0.5) |
| 7 | Coronary artery calcification |  | 5(0.5) |
| 8 | Atrial fibrillation |  | 5(0.5) |
| 9 | Hypertension |  | 106(10.6) |
| 10 | Heart failure |  | 3(0.3) |
| 11 | Coronary artery atherosclerosis |  | 5(0.5) |
| Digestive system diseases | | 139(13.9%) | 152(15.2) |
| 1 | Nonalcoholic fatty liver |  | 76(7.6) |
| 2 | Ulcerative colitis |  | 24(2.4) |
| 3 | Crohn’s disease |  | 8(0.8) |
| 4 | Hepatic fibrosis |  | 9(0.9) |
| 5 | Helicobacter pylori infection |  | 32(3.2) |
| 6 | Celiac disease |  | 3(0.3) |
| Endocrine diseases | | 298(29.8%) | 380(38.0) |
| 1 | Metabolic syndrome |  | 82(8.2) |
| 2 | Hashimoto’s thyroiditis |  | 22(2.2) |
| 3 | Diabetes mellitus |  | 50(5.0) |
| 4 | Obesity |  | 94(9.4) |
| 5 | Hyperlipidemia |  | 132(13.2) |
| Respiratory diseases | | 75(7.5%) | 75(7.5) |
| 1 | Obstructive sleep apnea |  | 33(3.3) |
| 2 | Sarcoidosis |  | 20(2.0) |
| 3 | Asthma |  | 14(1.4) |
| 4 | Chronic obstructive pulmonary disease |  | 8(0.8) |
| Urinary system diseases | | 54(5.4%) | 56(5.6) |
| 1 | IgA nephropathy |  | 16(1.6) |
| 2 | Glomerular diseases |  | 19(1.9) |
| 3 | Chronic kidney disease |  | 16(1.6) |
| 4 | End-stage renal disease |  | 5(0.5) |
| Hematological diseases | | 40(4.0%) | 40(4.0) |
| 1 | Autoimmune hemolytic anemia |  | 25(2.5) |
| 2 | Idiopathic thrombocytopenic purpura |  | 15(1.5) |
| Musculoskeletal diseases | | 60(6.0%) | 60(6.0) |
| 1 | Multiple osteomyelitis |  | 23(2.3) |
| 2 | Temporomandibular disease |  | 12(1.2) |
| 3 | Hyperactive leg syndrome |  | 16(1.6) |
| 4 | Fracture osteoporosis |  | 9(0.9) |
| Other skin diseases | | 123(12.3%) | 147(14.7) |
| 1 | Vitiligo |  | 13(1.3) |
| 2 | Contact dermatitis |  | 16(1.6) |
| 3 | Atopic dermatitis |  | 23(2.3) |
| 4 | Bullous pemphigus and pemphigoid |  | 5(0.5) |
| 5 | Pemphigus |  | 7(0.7) |
| 6 | Alopecia areata |  | 14(1.4) |
| 7 | Pruritus |  | 49(4.9) |
| 8 | Chronic spontaneous urticarial |  | 20(2.0) |
| Nervous system diseases | | 244(24.4%) | 342(34.2) |
| 1 | Depression |  | 114(11.4) |
| 2 | Guilt |  | 16(1.6) |
| 3 | Suicidal ideation |  | 6(0.6) |
| 4 | Insomnia |  | 87(8.7) |
| 5 | Burnout |  | 19(1.9) |
| 6 | Agitation or Irritability |  | 90(9.0) |
| Ocular manifestations | | 41(4.1%) | 41(4.1) |
| Malignant tumors | | 8(0.8%) | 8(0.8) |
